# Molecular Cloning and Functional Characterization of a Hexokinase from the Oriental River Prawn *Macrobrachium nipponense* in Response to Hypoxia

**DOI:** 10.3390/ijms18061256

**Published:** 2017-06-13

**Authors:** Shengming Sun, Fujun Xuan, Hongtuo Fu, Jian Zhu, Xianping Ge

**Affiliations:** 1 Key Laboratory of Genetic Breeding and Aquaculture Biology of Freshwater Fishes, Ministry of Agriculture, Freshwater Fisheries Research Center, Chinese Academy of Fishery Sciences, Wuxi 214081, China; sunsm@ffrc.cn (S.S.); gexp@ffrc.cn (X.G.); 2 Jiangsu Provincial Key Laboratory of Coastal Wetland Bioresources and Environmental Protection, Yancheng Teachers University, Yancheng 224051, China; xuanfujun@gmail.com

**Keywords:** hypoxia, hexokinase, hypoxia inducible factor, gene expression, *Macrobrachium nipponense*

## Abstract

Metabolic adjustment to hypoxia in *Macrobrachium nipponense* (oriental river prawn) implies a shift to anaerobic metabolism. Hexokinase (HK) is a key glycolytic enzyme in prawns. The involvement of HK in the hypoxia inducible factors (HIFs) pathway is unclear in prawns. In this study, the full-length cDNA for HK (MnHK) was obtained from *M. nipponense*, and its properties were characterized. The full-length cDNA (2385 bp) with an open reading frame of 1350 bp, encoded a 450-amino acid protein. MnHK contained highly conserved amino acids in the glucose, glucose-6-phosphate, ATP, and Mg^+2^ binding sites. Quantitative real-time reverse transcription PCR assays revealed the tissue-specific expression pattern of MnHK, with abundant expression in the muscle, and gills. Kinetic studies validated the hexokinase activity of recombinant HK. Silencing of HIF-1α or HIF-1β subunit genes blocked the induction of HK and its enzyme activities during hypoxia in muscles. The results suggested that MnHK is a key factor that increases the anaerobic rate, and is probably involved in the HIF-1 pathway related to highly active metabolism during hypoxia.

## 1. Introduction

Hexokinase (ATP: d-hexose-6-phosphotransferase, EC 2.7.1.1.) is the first key regulatory enzyme of the glycolytic pathway [1]. Hexokinases (HKs) are a family of conserved enzymes present in each domain of life, including bacteria, yeast, plants, and humans [2]. Several HK isoforms or isozymes providing different catalytic and regulatory properties can occur in a single species [3,4]. The most thoroughly studied are the four distinct hexokinase isozymes reported in mammals and named as types I–IV. Previous studies have demonstrated that hexokinase I, II, and III are 100 kDa proteins and exhibit marked sensitivity to inhibition by glucose-6-phosphate [5]. Hexokinase gene structure and function in crustaceans have been poorly studied compared with those of mammals. In crustaceans, lactate accumulates as an end product of anaerobic glycolysis during hypoxia [6,7].

Although four isozymes between 45 and 50 kDa (Hex-A, -B, -C and -T) were found at different stages of development and different tissues from the fruit fly *Drosophila melanogaster* [8], it is difficult to distinguish how many hexokinase isoenzymes exist in crustaceans. This may be because of tight linkage, disequilibrium, and post-translational modifications of hexokinases. Hexokinase activity has been found in the crab *Neohelice granulata* [9], the shrimp *Litopenaeus vannamei* [10], and the lobsters *Homarus americanus* and *Homarus vulgaris* [11]. Recently, multiple hexokinase gene sequences were identified in invertebrates, such as the sea squirt *Ciona intestinalis* [12], the parasitic nematode *Brugia malayi* [13], the parasite *Trypanosoma cruzi* [14], and the shrimp *Litopenaeus vannamei* [6]. However, little information is available regarding the expression pattern and activity of HKs in the oriental river prawn *Macrobrachium nipponense* (Crustacea; Decapoda; Palaemonidae). *M. nipponense*, an important commercial prawn species in China, is an interesting hypoxia model because of its susceptibility to hypoxia compared with most other crustaceans [15]. Low dissolved oxygen conditions lead to hypoxia, which causes catastrophic mortality events in prawns. Therefore, for the sustained development of prawn aquaculture, a deeper understanding of the energy metabolism mechanism induced by hypoxia in *M. nipponense* is essential. 

Considering that mammalian HK expression is induced by the transcription factor hypoxia inducible factor 1 (HIF-1) [16,17], we hypothesized that MnHK expression would be regulated during hypoxia via an HIF-1-dependent mechanism. In this study, we aimed to identify and characterize the hexokinase gene in *M. nipponense* and to compare its expression levels in response to hypoxia after silencing the α and β subunits of HIF-1, using RNA interference.

## 2. Results and Discussion

### 2.1. Characteristics and Phylogeny of MnHK

**Figure 1:**
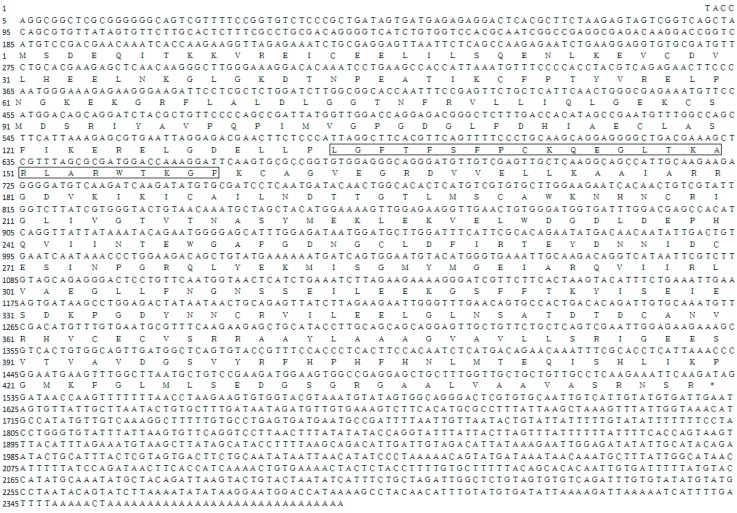
Nucleotide and predicted amino acid sequences for *Macrobrachium nipponense* hexokinase (HK) cDNA. HK signature sequences are shown in boxes. The asterisk indicates the stop codon.

**Figure 2:**
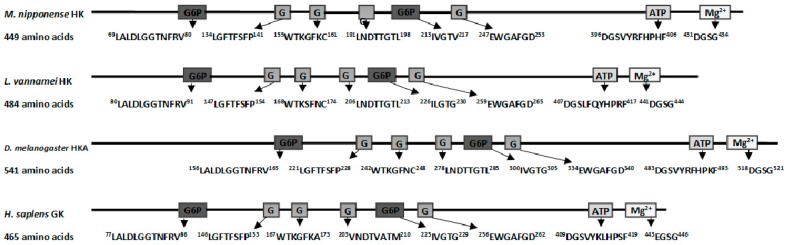
Graphical representation of hexokinase (HK) domains from *Macrobrachium nipponense* (HK, KY270495), *Litopenaeus vannamei* (HK, ABO21409), *Drosophila melanogaster* (HKA-A, NP_524848), and *Homo sapiens* (GK 1, AAB97682). The amino acids implicated in glucose (G), glucose-6-phosphate (G6P), ATP, and Mg2+ binding sites are indicated in boxes.

**Figure 3:**
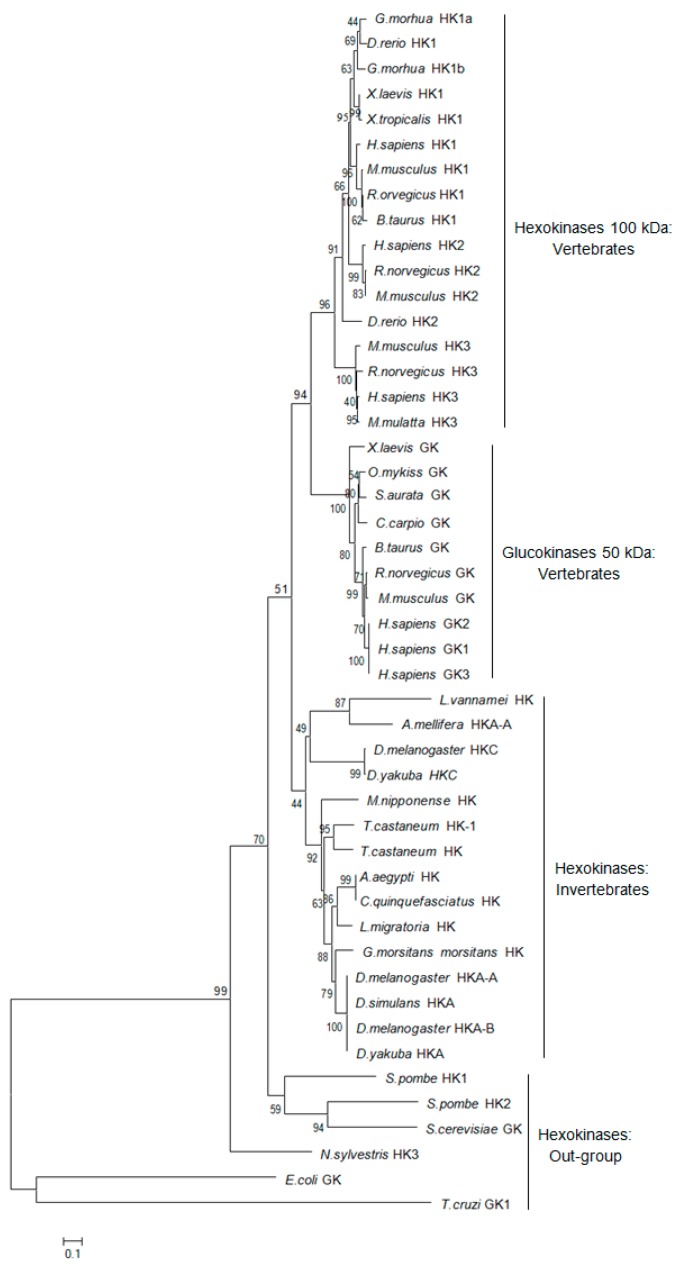
Phylogenetic tree based on the alignment of known amino acid sequences of hexokinase proteins. The numbers shown at the branches indicate the bootstrap values (%). Sequences used in the analysis with their abbreviation and GenBank accession number were as follows: Hexokinase *Macrobrachium nipponense*, (KY270495; this work); Hexokinases 1: *Gadus morhua* HK1a (ABS89272), *G. morhua* HK1b (ABS89273), *Danio rerio* (AAH67330), *Schizosaccharomyces pombe* (CAA90848), *Xenopus laevis* (NP_001096656), *Xenopus tropicalis* (NP_001096201), *Homo sapiens* (AAH08730), *Rattus norvegicus* (NP_036866), *Mus musculus* (NP_001139572), and *Bos taurus* (AAA51661). Hexokinases 2: *H. sapiens* (AAH21116), *R. norvegicus* (NP_036867), *M. musculus* (AAH54472), *D. rerio* (AAH45496), and *S. pombe* HK2 (CAA63488). Hexokinase 3: *M. musculus* (NP_001028417), *R. norvegicus* (NP_071515), *H. sapiens* (NP_002106), *Macaca mulatta* (XP_001086179), and *Nicotiana sylvestris* (AAT77513). Invertebrate hexokinases: *D. melanogaster* (HKA-A (AAF46507), HKC (AAF58160), and HKA-B (AAN09253)), *D. simulans* (HKA (ABW93133) and HKC (EDX07186)), *D. yakuba* (HKA (EDX02859) and HKC (EDW91124)), *Apis mellifera* HKA-A (XP_392350), *Tribolium castaneum* (HK (XP_970645) and (XP_966410)), *Aedes aegypti* HK (AAU05129), *Locusta migratoria* (ACM78948), *Glossina morsitans morsitans* (ADD20426), and *Culex quinquefasciatus* (XP_001850122). Glucokinases: *X. laevis* (AAI70499), *Oncorhynchus mykiss* (NP_001117721), *Sparus aurata* (AAC33585), *H. sapiens* (GK1 (NP_000153), GK2 (NP_277042), and GK3 (NP_277043)), *Cyprinus carpio* (AAC33587), *B. taurus* (NP_001095772), *R. norvegicus* (NP_036697), *M. musculus* (NP_034422), *Saccharomyces cerevisiae* (AAA53536), *Escherichia coli* (AP_002988), and *Trypanosoma cruzi* GK1 (XP_821474).

**Figure 4:**
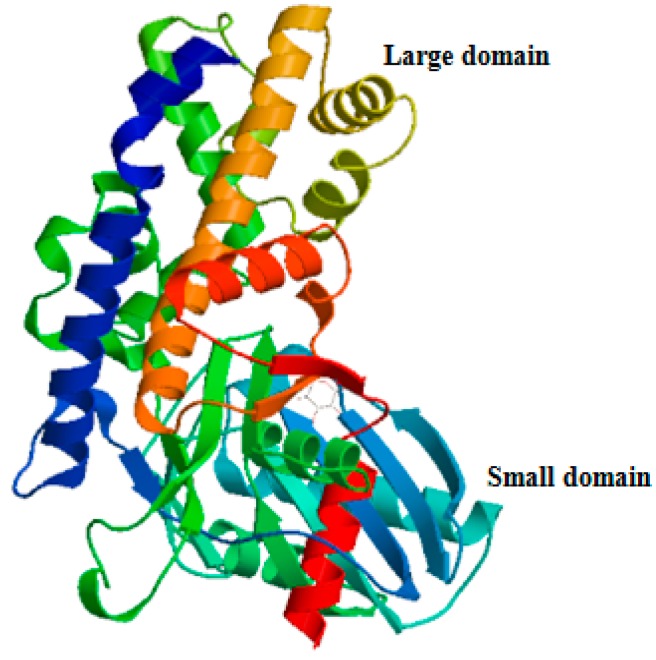
Three-dimensional (3D) structures of *M. nipponense* hexokinase (HK) predicted using human hexokinases II as template models. Different secondary structure was marked in different colors.

Rapid amplification of cDNA ends (RACE) of the HK fragment yielded a cDNA sequence of 2385 bp (GenBank Accession No. KY270495). The cDNA sequence has a predicted a start codon at nucleotide 185 and a stop codon at nucleotide 1532. The deduced open reading frame of 1350 bp encodes a putative protein of 450 amino acid residues (Figure 1) with an estimated molecular mass of 49.72 kDa and a predicted isoelectric point of 5.29, which is similar to invertebrate hexokinases and mammalian type IV HKs [3]. Sequence comparison of the MnHK protein with HK proteins from other species identified conserved amino acids in the glucose, glucose-6-phosphate, and ATP binding domains (Figure 2), which was consistent with previous reports that glycolytic enzymes are considered as the most ancient and highly conserved proteins and DNA sequences among several organisms [18,19,20]. In the phylogenetic tree, MnHK was positioned as a separate branch at the base of invertebrate HKs and was separated from vertebrate hexokinases (Figure 3), which was in agreement with the traditional taxonomy of the included species. This was further supported by three-dimensional (3D)-modeling, which showed that MnHK has a tertiary structure that shares many features typical of hexokinases, including the core of a β-pleated sheet and the surrounding helix (Figure 4).

### 2.2. Tissue-Specific Expression of MnHK

**Figure 5:**
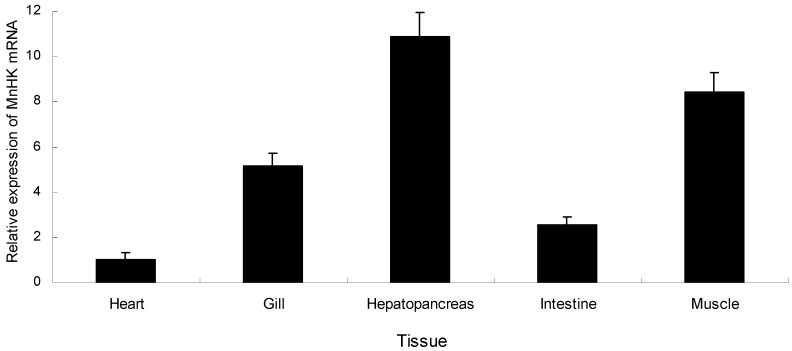
Quantitative real-time reverse transcription PCR (RT-qPCR) analysis of hexokinase gene expression in various tissues of *M. nipponense*. The ratio refers to the gene expression in different tissues compared with that in the heart. The β-actin gene was used as an internal control, as previously described, and MnHK mRNA expression in other tissues was normalized to the expression level in heart tissue. Vertical bars represent mean ± standard error of the mean (SEM) values for triplicate samples.

Quantitative real-time reverse transcription PCR (qRT-PCR) was used to examine the expression pattern of *MnHK* in the different tissues of *M. nipponense*, including the hepatopancreas, intestine, heart, muscle, and gills. *MnHK* mRNA was constitutively expressed in all the examined tissues, with higher expression in the muscle and hepatopancreas (Figure 5), which was similar to previous studies in chicken [21], mouse [22], and fish [23]. Hepatopancreas functions include the production of digestive enzymes and hemolymph proteins, and the absorption of nutrients [24], while anaerobic respiration mainly occurs in muscles with functions in locomotion and gluconeogenesis [25]. It is reasonable to think that glycolysis would occur in certain cells of the hepatopancreas and muscle, which would explain the high level of *MnHK* transcripts in the hepatopancreas and muscle.

### 2.3. Expression and Purification of Recombinant MnHK

**Figure 6:**
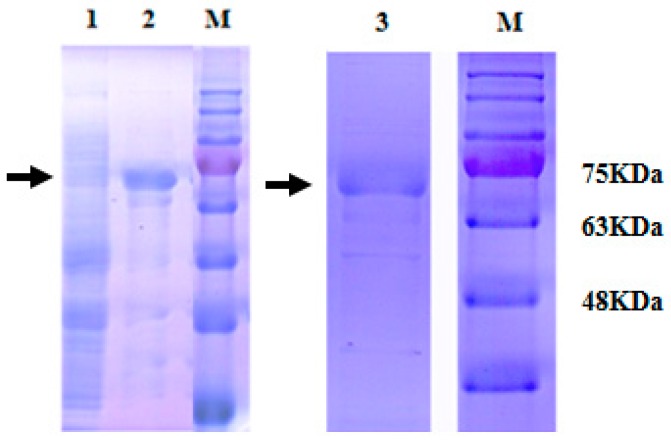
Sodium dodecyl sulfate-polyacrylamide gel electrophoresis (SDS-PAGE) analysis of the purified recombinant *M. nipponense* hexokinase protein (rMnHK). M: molecular mass standards; lane 1: *Escherichia coli* crude extract, without induction; lane 2: induced expression for 2 h of rMnHK; lane 3: purified protein collected at the elution step. The running positions of molecular mass standards are indicated on the left.

Expression of pET-28a-MnpHK was induced with 1 mM isopropyl-β-d-galactopyranoside (IPTG) for 2 h at 30 °C. After sonication and centrifugation, the bacterial supernatants were analyzed using sodium dodecyl sulfate-polyacrylamide gel electrophoresis (SDS-PAGE). The subunit molecular weight of the recombinant MnHK protein (rMnHK) was approximately 70 kDa, which corresponded to the molecular weight of MnHK plus the His tag from the vector (Figure 6). The recombinant protein was further purified to homogeneity using affinity column chromatography (Figure 6). The molecular weight of rMnHK was similar to plant [26,27,28,29,30] and animal [8,23] hexokinases.

### 2.4. Kinetic Characterization of rMnHK

One key feature of the different HK isozymes is their affinity for glucose, which in humans, changes by one order of magnitude in the order type III > type I > type II [4]. Thus, biochemical characterization of rMnHK was performed to validate its HK activity and to explain its sensitivity to inhibition by Gluc-6-P. The pH optimum of rMnHK was determined as 8.45, and the enzyme’s activity followed Michaelis–Menten kinetics with respect to substrate concentrations. The recombinant enzyme could use glucose, fructose, mannose, and galactose as substrates. The Km values for different substrates are shown in (Table 1) and were similar to other invertebrate hexokinases, such as those from *Schistosoma mansoni* [31], *Trypanosoma cruzi* [3], and *Trypanosoma brucei* [32]. The low Km observed for glucose was similar to that of mammalian hexokinase isoenzymes I, II, and III, as well as other invertebrate hexokinases [5,14].

### 2.5. MnHK Expression and Activity in Muscles of Prawns During Hypoxia

**Figure 7:**
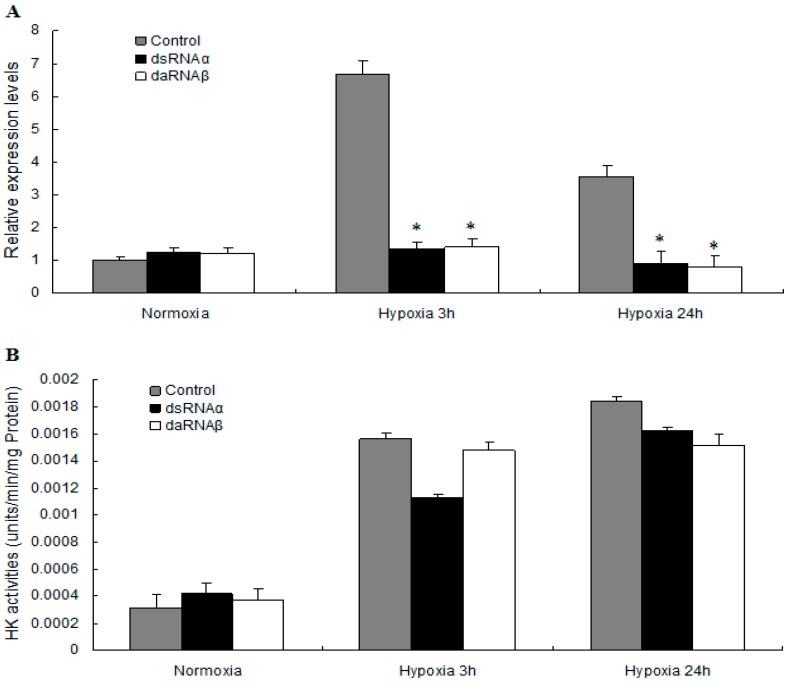
Hexokinase mRNA expression (**A**) and enzyme activity (**B**) in muscles during hypoxia. Prawns were exposed to hypoxia (2.0 mg/L dissolved oxygen (DO)) for 3 and 24 h and injected with saline solution (control) or HIF-1-dsRNA. Double-stranded (ds)RNAα and dsRNAβ labels indicate animals injected with the corresponding dsRNA for HIF-1α and HIF-1β gene silencing, respectively. One-way ANOVA was used to compare the experimental treatments. (*) indicates significant differences (*p* < 0.05) between the control and dsRNAα groups, between the control and dsRNAβ, respectively. Vertical bars represent mean ±SEM values for triplicate samples.

Hypoxia inducible factors (HIFs) are a family of transcription factors that induce the expression of several hundred genes directly in animal cells [33]. In prawns silenced for either the α or β subunits of HIF-1, the expression of HK mRNA in the muscle was relatively low in the hypoxia group, but relatively high in the non-silenced control group. The MnHK mRNA level increased by 6.65-fold in the muscles from prawns of the non-silenced control group after 3 h of hypoxia compared to normoxia, and slowly decreased after 24 h of hypoxia. In contrast, this increase in HK expression was abolished when HIF-1 is absent, indicating that HK expression is regulated by HIF-1 under hypoxic conditions (Figure 7A). Our results provided evidence that HK is induced by hypoxia and, accompanied by the previous finding that lactate accumulated in prawns subjected to hypoxia [7], suggested that HK could be a key factor that accelerates the rate of glycolysis to produce ATP anaerobically.

Variations of in HK activity have been observed in crustaceans under different environmental conditions, such as *Carcinus maenas* during the moult cycle [33], *Litopenaeus vannamei* in different salinity levels [34], and *L. vannamei* during the moult cycle [10]. A similar pattern of variations was found in the present study: HK activity increased 4.88-fold and 5.78-fold in muscles from prawns exposed to hypoxia for 3 and 24 h, respectively, compared with the normoxic controls. No significant changes in HK activity were detected in the HIF-1α or HIF-1β-silenced prawns in response to hypoxia for 3 and 24 h; however, prawns from the HIF-1α or HIF-1β silenced group had higher HK activity after 3 and 24 h of hypoxia compared with the normoxia group (Figure 7B). In contrast to the data for the HK mRNA levels, these results demonstrated that despite silencing of HIF-1, HK activity increased in the muscles of prawns under hypoxic conditions. This lack of correlation between mRNA levels and enzyme activity has been described in several rat tissues [35], fish tissues [36], and shrimp tissues [6]. A reasonable explanation is that the differences in HK expression and activity could be related to the presence of isozymes in the prawns. Further study is needed to determine the subunit compositions and expression regulation of HK isozymes.

Although it is well known that HIF-1 recognizes hypoxia response elements (HRE) present in the promoter regions of genes encoding glycolytic enzymes such as aldolase A, Enolase 1, lactate dehydrogenase (LDH) A, HK II, glucose transport 1, and pyruvate dehydrogenase in mammals induced by hypoxia [16,17,37], little information about HK gene expression under hypoxic stress in crustaceans has been reported. The RNAi results showed that HIF-1 upregulated HK gene expression in the muscles of prawns. The promoter region of the *MnHK* gene has not been identified; therefore, the details of the role of HIF-1 in the regulation of *MnHK* expression should be determined in a future study.

## 3. Materials and Methods

### 3.1. Ethics Statement

All animals and experiments were conducted in accordance with the “Guidelines for Experimental Animals” of the Ministry of Science and Technology (Beijing, China). All efforts were made to minimize suffering. The animal procedures were performed in accordance with the standard set out in the Guidelines for the Care and Use Committee of the Chinese Academy of Fishery Sciences. The study protocols (FFRC125) were approved by the Institute for Experimental Animals of Chinese Academy of Fishery Sciences on 26 August 2016.

### 3.2. Experimental Animals and Hypoxia Treatment

Three hundred healthy oriental river prawns, with wet weights of 1.76–2.68 g, were obtained from Tai lake in Wuxi, China (120°13′44″ E, 31°28′22″ N). The prawns were transported to the laboratory and maintained in six 300-liter tanks with aerated freshwater for one week to acclimatize them to their new environment. In the hypoxia challenge trial, 240 prawns were divided randomly into two groups in triplicate, and maintained in filtered fresh water. A control group of prawns was maintained under normoxic conditions (6.5 ± 0.2 mg O_2_ L^−1^). In the hypoxia group, hypoxic conditions (2.0 ± 0.1 mg O_2_ L^−1^) within the treatment tanks were maintained for 3 and 24 h by bubbling nitrogen into the tank, as described previously [7]. All exposures were conducted in triplicate for both the control and treatment groups. At each time point, three prawns were removed in each of the three tanks for each group. Approximately 100 mg of hepatopancreas, gills, muscle, intestine, and heart from healthy prawns were immediately excised and frozen in liquid nitrogen, and then stored at −80 °C for subsequent study. 

### 3.3. Cloning of the MnHK cDNAs

Total RNA from mixed tissues of *M. nipponense* was extracted using RNAiso Plus Reagent (Takara, Tokyo, Japan). First strand cDNA was synthesized using a reverse transcriptase M-MLV Kit (Takara). 3′ and 5′RACE were performed using a 3′-full RACE Core Set Ver.2.0 Kit and a 5′-full RACE Kit (Takara) to determine the cDNA 3′ and 5′ ends of *MnHK*. All the primers designed from obtained partial cDNA sequences were used in the cloning are listed in Table 2. The purified products were cloned into the pMD18-T vector (Takara) and sequenced by ABI3730 DNA analyzer.

### 3.4. Nucleotide Sequence and Bioinformatics Analyzes

Amino acid sequences were deduced using the ORF Finder program (Available online: http://ncbi.nlm.nih.gov/gorf/gorf.html). The sequences were analyzed employing the BLASTX and BLASTN programs (Available online: http://www.ncbi.nlm.nih.gov/BLAST/) in the nucleotide and protein databases at NCBI. Multiple sequence alignments of *MnHK* were carried out using the Clustal W Multiple Alignment program (Available online: http://www.ebi.ac.uk/clustalw/). Phylogenetic trees were generated using the neighbor-joining method in Molecular Evolutionary Genetics Analysis software version 4.0 (Available online: http://www.megasoftware.net/mega4/mega.html). The 3D model of *M. nipponense* HK was predicted using fully-automated protein structure homology modeling (Available online: http://www.expasy.org/swissmod/SWISSMODEL.html), using human hexokinase II (PDB code: 2nztA) as the model.

### 3.5. HIF-1 Silencing and Hypoxia

Double-stranded RNA (dsRNA) of *MnHIF-1α* (KP050352) and *MnHIF-1β* (KP050353) were synthesized in vitro using a Transcript AidTM T7 High Yield Transcription kit (Fermentas Inc., Waltham, MA, USA), according to the manufacturer’s instructions. Nucleotides 152–1484 of the *MnHIF-1α* cDNA and 140–1074 of the *MnHIF-1β* cDNA from *M. nipponense* were used for double-stranded (ds)RNA synthesis [38]. The purity and integrity of the dsRNA were examined by standard agarose gel electrophoresis. Ten prawns were injected intramuscularly with a dose of 4 μg/g of body weight (α or β, separately) as described previously [39]. The injected prawns were then subjected to normoxia (6.5 mg/L O_2_) for 24 h and hypoxia (2.0 mg/L O_2_) for 1, 3, and 24 h. A group of 10 control prawns were injected with saline solution and subjected to the same normoxic and hypoxic conditions. Four prawns from each group were collected randomly and dissected; muscle tissues were immediately frozen in liquid nitrogen and stored at −80 °C for further analysis.

### 3.6. qRT-PCR Analysis of MnHK Expression

The mRNA levels of *MnHK* in different tissues and under hypoxia conditions were detected by qRT-PCR. The cDNAs from different tissues and hypoxia treatments of *M. nipponense* were synthesized from total DNA-free RNA using a Prime Script RT reagent kit (Takara) following the manufacturer’s instructions. qRT-PCR was executed on a Bio-Rad iCycler iQ5 Real-Time PCR system (Bio-Rad, Hercules, CA, USA) using the designed primers (Table 1). The PCR reactions were conducted as described previously [40]. The relative quantification of the target and reference genes were evaluated based on the standard curve method. The expression levels of *MnHK* mRNA were calculated using the 2^−ΔΔ*C*t^ method [41].

### 3.7. Expression, Purification of MnHK and Antibody Production

*MnHK* cDNA was subcloned between the EcoRI and HindIII sites of the pET-28a vector (Novagen, Darmstadt, Germany) using PCR. A colony containing pET-28a with the target clone in the correct orientation was identified using restriction enzyme digestion, and the DNA sequence of the clone was confirmed by sequencing. Overexpression of the recombinant protein was performed according to the manufacturer‘s instructions (Novagen). *Escherichia coli* BL21 (DE3) containing the plasmid were inoculated into 500 mL of lysogeny broth containing ampicillin (50-mg/mL) and incubated with vigorous shaking until the OD_600_ value was 0.6. Isopropyl β-d-1-thiogalactopyranoside was then added to a final concentration of 0.8 mM, and the culture was incubated for 3 h. The cells were collected by centrifugation and resuspended in 100 mL of 1× binding buffer (5 mM imidazole/0.5 mM NaCl/20 mM Tris-HCl, pH 7.9), and broken by three rounds of sonication for 15 s each at 50% maximum power (Branson Sonifier 250; Branson, MO, USA). The insoluble fraction containing recombinant *MnHK* was collected by centrifugation and resuspended in 100 mL of binding buffer by sonication. The sample was maintained on ice for 1 h and centrifuged at 38,000× *g* for 20 min. The recombinant MnHK (rMnHK) was purified by using His-Tag system (Novagen) according to the manufacturer’s instructions, and was subjected to sodium dodecyl sulfate (SDS)-polyacrylamide gel electrophoresis (PAGE). The purified rMnHK was used to produce rabbit anti-HK antibodies as previous described [42].

### 3.8. Kinetic Investigations

The Km values for glucose, fructose, and ATP of rMnHK were determined by plotting the reaction rates against substrate concentration to fit the Michaelis–Menten equation. The effect of pH on rMnHK activity was measured using Tris–HCl buffer over a pH range of 7.0 to 8.8. The effects of various inhibitors, such as mannoheptulose, ADP, glucosamine, *N*-acetyl glucosamine, PPi, glucose-6-phosphate, *p*-chloromercuribenzoate (pCMB), *N*-ethylmaleimide (NEM); and metal chelators like *O*-phenanthrolene and ethylenediaminetetraacetic acid (EDTA), were studied by incubating the enzyme with each inhibitor for 10 min at 25 °C.

### 3.9. Enzymatic Activity Assay

Muscle tissue (100 mg) was homogenized in four volumes (*w*/*v*) of 0.1 M Tris-HCl, 5 mM β-mercaptoethanol, pH 8. The homogenate was centrifuged at 8000× *g* for 15 min at 4 °C, and the aqueous phase was precipitated by adding eight volumes of acetone. The acetone precipitate (2 mg) was reconstituted in 120 μL of Tris.MgCl_2_ buffer (0.05 M Tris HCl, 13.3 mM MgCl_2_, pH 8) to measure HK activity and was termed the crude extract. The enzyme activity was measured by mixing 230 μL of Tris.MgCl_2_ buffer, 50 μL of 0.67 M glucose, 10 μL of 16.5 mM ATP, 10 μL of 6.8 mM NAD, and 1 μL of glucose-6-phosphate dehydrogenase (GPDH, 300 U/mL Sigma, Shanghai, China). After incubation at 30 °C for 6–8 min, the reaction was started by adding 20 μL of the crude extract. HK activity was reported as micromoles of NADH formed per minute at 30 °C per milligram of protein. All measurements were done in triplicate and the method was adapted for use in microplates, for which the absorbance at 340 nm was measured in a SpectraMax M2 (Molecular Devices, Sunnyvale, CA, USA). A negative control without glucose was included.

### 3.10. Statistical Analysis

All data are presented as the mean ± SEM (standard error of the mean; *n* = 3). A Student‘s *t*-test was used to identify significant differences in *MnHK* gene expression between the controls and the tested samples using SPSS 15.0 software. A *p* value ≤ 0.05 was considered statistically significant.

## 4. Conclusions

In summary, this study demonstrated that hypoxia-induced HK gene expression is regulated in a HIF-1 dependent manner, and that this cellular response is oriented to ensure the contribution of HK to accelerate the rate of glycolysis in order to generate energy. This study would improve our understanding of the energy metabolic mechanism for prawns in response to hypoxia stress.

## Figures and Tables

**Table 1:** Kinetic characteristics of recombinant *M. nipponense* hexokinase.

Optimum pH	Kinetic Constant (Km) (mM)			Inhibitor Studies (K_i_) (mM)		
	Glucose	Fructose	ATP	ADP	PPi	G6P
8.46	0.045 ± 0.005	79 ± 0.6	0.88 ±0.6	0.76	0.011	N.I.

A Lineweaver–Burk plot was used to determine the Km and Ki of *M. nipponense* hexokinase. The number of determinations was three in each case and values are mean of three experiments. N.I.: No inhibition.

**Table 2:** List of primers used in this study.

Primer	Primer Sequence (5′–3′)
MnHK-F1 (5′RACE out primer)	CAAGAAGAGGGGATGTCAAG
MnHK-F2 (5′RACE in primer)	GTTTCCCCACCTACGTCAGA
MnHK-R1 (3′RACE out primer)	GGGATGTTGTCGAGTTGCTC
MnHK-R2 (3′RACE in primer)	GACGGGCTCTTTGACCACAT
MnHK-F (Real-time PCR primer)	GGGATGTTGTCGAGTTGCTC
MnHK-R (Real-time PCR primer)	TCGTCCAAATCACCATCCCA
MnpHK CDS amplification (*Nde*I)	CGAGGCGAGACAAGGACCGGTCATG
MnpHK CDS amplification (*Xho*I)	ATCCTATCTTGAATTTCTTGAGGC
β-Actin F (Real-time PCR primer)	TATGCACTTCCTCATGCCATC
β-Actin R (Real-time PCR primer)	AGGAGGCGGCAGTGGTCAT

## Abbreviations

HK	Hexokinase
qRT-PCR	Quantitative real-time reverse transcription PCR
RACE	Rapid amplification of cDNA ends
HIF	Hypoxia inducible factor
3D	Three-dimensional
DO	Dissolved oxygen
HRE	Hypoxia response elements
SDS	Sodium dodecyl sulfate
IPTG	Isopropyl-β-d-galactopyranoside
dsRNA	Double-stranded RNA
GPDH	Glucose-6-phosphate dehydrogenase
ATP	Adenosine triphosphate
EDTA	Ethylenediaminetetraacetic acid

## References

[1] Tsai H.J., Wilson J.E. (1997). Functional organization of mammalian hexokinases: Characterization of the rat type III isozyme and its chimeric forms, constructed with the N- and C-terminal halves of the type I and type II isozymes. Arch. Biochem. Biophys..

[2] Iynedjian P.B. (1993). Mammalian glukokinase and its gene. Biochem. J..

[3] Cárdenas M.L., Cornish-Bowden A., Ureta T. (1998). Evolution and regulatory role of the hexokinases. Biochim. Biophys. Acta.

[4] Wilson J.E. (2003). Isozymes of mammalian hexokinase: Structure, subcellular localization and metabolic function. J. Exp. Biol..

[5] Wilson J.E. (1995). Hexokinases. Reviews of Physiology, Biochemistry and Pharmacology.

[6] Soñanez-Organis J.G., Rodriguez-Armenta M., Leal-Rubio B., Peregrino-Uriarte A.B., Gómez-Jiménez S., Yepiz-Plascencia G. (2012). Alternative splicing generates two lactate dehydrogenase subunits differentially expressed during hypoxia via HIF-1 in the shrimp *Litopenaeus vannamei*. Biochimie.

[7] Sun S.M., Xuan F.J., Ge X.P., Fu H.T., Zhu J., Zhang S.Y. (2014). Identification of differentially expressed genes in hepatopancreas of oriental river prawn, *Macrobrachium nipponense* exposed to environmental hypoxia. Gene.

[8] Jayakumar P.C., Shouche Y.S., Patole M.S. (2001). Cloning of two hexokinase isoenzyme sequences from *Drosophila melanogaster*. Insect Biochem. Mol. Biol..

[9] Lauera M.M., de Oliveira C.B., Yano N.L.I., Bianchinia A. (2012). Copper effects on key metabolic enzymes and mitochondrial membrane potential in gills of the estuarine crab *Neohelice granulata* at different salinities. Comp. Biochem. Physiol. C.

[10] Gaxiola G., Cuzon G., Garcia T., Taboada G., Brito R., Chimal M.E., Paredes A., Soto L., Rosas C., van Wormhoudt A. (2005). Factorial effects of salinity, dietary carbohydrate and moult cycle on digestive carbohydrases and hexokinases in *Litopenaeus vannamei* (Boone, 1931). Comp. Biochem. Physiol. A.

[11] Stetten M. R., Goldsmith P.K. (1981). Two hexokinases of *Homarus americanus* (lobster), one having great affinity for mannose and fructose and low affinity for glucose. Biochim. Biophys. Acta.

[12] Irwin D.M., Tan H. (2008). Molecular evolution of the vertebrate hexokinase gene family: Identification of a conserved fifth vertebrate hexokinase gene. Comp. Biochem. Physiol. D.

[13] Singh A.R., Joshi S., Arya R., Kayastha A.M., Srivastava K.K., Tripathi L.M., Saxena J.K. (2008). Molecular cloning and characterization of *Brugia malayi* hexokinase. Parasitol. Int..

[14] Cáceres A.J., Portillo R., Acosta H., Rosales D., Quiñones W., Avilan L., Salazar L., Dubourdieu M., Michels P.A., Concepción J.L. (2003). Molecular and biochemical characterization of hexokinase from *Trypanosoma cruzi*. Mol. Biochem. Parasitol..

[15] Guan Y.Q., Li L., Wang H.C., Wang Z.L. (2010). Effects of hypoxia on respiratory metabolism and antioxidant capability of *Macrobrachium nipponense*. J. Hebei Univ..

[16] Semenza G.L., Jiang B.H., Leung S.W., Passantino R., Concordet J.P., Mair P., Giallongo A. (1996). Hypoxia response elements in the aldolase A, enolase 1, and lactate dehydrogenase A gene promoters contain essential binding sites for hypoxia-inducible factor 1. J. Biol. Chem..

[17] Riddle S.R., Ahmad A., Ahmad S., Deeb S.S., Malkki M., Schneider B.K., Allen C.B., White C.W. (2000). Hypoxia induces hexokinase II gene expression in human lung cell line A549. Am. J. Physiol. Lung Cell Mol. Physiol..

[18] Lonberg N., Gilbert W. (1985). Intron/exon structure of the chicken pyruvate kinase gene. Cell.

[19] Peak M.J., Peak J.G., Stevens F.J., Blamey J., Mai X., Zhou Z.H., Adams M.W.W. (1994). The hyperthermophilic glycolytic enzyme enolase in the archaeon, Pyrococcus furiosus: Comparison with mesophilic enolases. Arch. Biochem. Biophys..

[20] Webster K.A. (2003). Evolution of the coordinate regulation of glycolytic enzyme genes by hypoxia. J. Exp. Biol..

[21] Seki Y., Stao K., Kono T., Akiba Y. (2005). Cloning and gene expression of hexokinase I and II in the chicken skeletal muscle. Anim. Sci. J..

[22] Heikkinen S., Suppola S., Malkki M., Deeb S.S., Jänne J., Laakso M. (2000). Mouse hexokinase II gene: Structure, cDNA, promoter analysis, and expression pattern. Mamm. Genome.

[23] Li M.Y., Gao Z., Wang Y., Wang H., Zhang S.C. (2014). Identification, expression and bioactivity of hexokinase in amphioxus: Insights into evolution of vertebrate hexokinase genes. Gene.

[24] Yepiz-Plascencia G., Gollas-Galvan T., Vargas-Albores F., Garcia-Bañuelos M. (2000). Synthesis of hemolymph high-Density Lipoprotein beta-Glucan binding protein by Penaeus vannamei shrimp hepatopancreas. Mar. Biotechnol..

[25] Cota-Ruiz K., Peregrino-Uriarte A.B., Felix-Portillo M., Martínez-Quintana J.A., Yepiz-Plascencia G. (2015). Expression of fructose 1,6-bisphosphatase and phosphofructokinase is induced in hepatopancreas of the white shrimp Litopenaeus vannamei by hypoxia. Mar. Environ. Res..

[26] Renz A., Merlo L., Stitt M. (1993). Partial purification from potato tubers of three fructokinases and three hexokinases which show differing organ and developmental specificity. Planta.

[27] Miernyk J.A., Dennis D.T. (1983). Mitochondrial, plastid and cytosolic isozymes of hexokinase from developing endosperm of Ricinus communis. Arch. Biochem. Biophys..

[28] Yamamoto Y.T., Prata R.T.N., Williamson J.D., Weddington M., Pharr D.M. (2000). Formation of a hexokinase complex is associated with changes in energy utilization in celery organs and cells. Physiol. Plant..

[29] Troncoso-Ponce M.A., Rivoal J., Dorion S., Moisan M.C., Garcés R., Martínez-Force E. (2011). Cloning, biochemical characterization and expression of a sunflower (*Helianthus annuus* L.) hexokinase associated with seed storage compounds accumulation. J. Plant Physiol..

[30] Zhao J., Sun M.H., Hu D.G., Hao Y.J. (2016). Molecular Cloning and Expression Analysis of a Hexokinase Gene, MdHXK1 in Apple. Hortic. Plant J..

[31] Tielens A.G., van den Heuvel J.M., van Mazijk H.J., Wilson J.E., Shoemaker C.B. (1994). The 50-kDa glucose 6-phosphate-sensitive hexokinase of *Schistosoma mansoni*. J. Biol. Chem..

[32] Misset O., Bos O.J., Opperdoes F.R. (1986). Glycolytic enzymes of *Trypanosoma brucei:* Simultaneous purification, intraglycosomal concentrations and physical properties. Eur. J. Biochem..

[33] Loret S.M., Devos P.E. (1992). Hydrolysis of G-6P by a microsomal specific phosphatase and glucose phosphorylation by a low Km hexokinase in the digestive gland of the crab *Carcinus maenas*. Variations during the moult cycle. J. Comp. Physiol. B.

[34] Rosas C., Cuzon G., Gaxiola G., Le Priol Y., Pascual C., Rossignyol J., Contreras F., Sa´nchez A., van Wormhoudt A. (2001). Metabolism and growth of juveniles of *Litopenaeus vannamei*: Effect of salinity and dietary carbohydrates level. J. Exp. Mar. Biol. Ecol..

[35] Rossignol F., Solares M., Balanza E., Coudert J., Clottes E. (2003). Expression of lactate dehydrogenase A and B genes in different tissues of rats adapted to chronic hypobaric hypoxia. J. Cell. Biochem..

[36] Yang T.H., Somero G.N. (1996). Activity of lactate dehydrogenase but not its concentration of messenger RNA increases with body size in barred sand bass, *Paralabrax nebulifer* (Teleostei). Biol. Bull..

[37] McClelland G.B., Brooks G.A. (2002). Changes in MCT 1, MCT 4, and LDH expression are tissue specific in rats after long-term hypobaric hypoxia. J. Appl. Physiol..

[38] Sun S.M., Xuan F.J., Fu H.T., Ge X.P., Zhu J., Qiao H., Jin S.B., Zhang W.Y. (2016). Molecular characterization and mRNA expression of hypoxia inducible factor-1 and cognate inhibiting factor in *Macrobrachium nipponense* in response to hypoxia. Comp. Biochem. Physiol. B.

[39] Bai H.K., Qiao H., Li F.J., Fua H.Y., Jiang S.F., Zhang W.Y., Yan Y.D., Xiong Y.W., Sun S.M., Jin S.B. (2016). Molecular and functional characterization of the vitellogenin receptor in oriental river prawn, *Macrobrachium nipponense*. Comp. Biochem. Physiol. A.

[40] Qiao H., Xiong Y.W., Zhang W.Y., Fu H.G., Jiang S.F., Sun S.M., Bai H.K., Jin S.B., Gong Y.S. (2015). Characterization, expression, and function analysis of gonad-inhibiting hormone in Oriental River prawn, *Macrobrachium nipponense* and its induced expression by temperature. Comp. Biochem. Physiol. A.

[41] Livak K.J., Schmittgen T.D. (2001). Analysis of relative gene expression data using realtime quantitative PCR and the 2^−ΔΔ*C*t^ method. Methods.

[42] Sun S.M., Gu Z.M., Fu H.T., Zhu J., Ge X.P., Xuan F.J. (2016). Molecular cloning, characterization, and expression analysis of p53 from the oriental river prawn, *Macrobrachium nipponense*, in response to hypoxia. Fish Shellfish Immunol..

